# Case report: Treatment of non-medical tetrahydrocannabinol toxicosis with transmucosal cannabidiol-infused dissolving sheets in six dogs

**DOI:** 10.3389/fvets.2024.1448123

**Published:** 2024-12-09

**Authors:** Kyra Marsigliano, Katie Green, Brian A. DiGangi

**Affiliations:** ^1^Intracoastal West Veterinary Hospital, Jacksonville, FL, United States; ^2^Department of Small Animal Clinical Sciences, College of Veterinary Medicine, University of Florida, Gainesville, FL, United States

**Keywords:** canine, marijuana toxicosis, THC, CBD, transmucosal

## Abstract

Increased cases of canine tetrahydrocannabinol (THC) toxicosis have been reported in North America in recent years. Cases are often evaluated on an emergency basis and treatment has relied upon supportive care which can be costly and prohibitive for some pet owners. The purpose of this report is to describe the clinical findings and outcomes in dogs with non-medical, presumptive THC toxicosis treated by administration of a cannibidiol (CBD)-infused transmucosal dissolving sheet. Medical records of six cases of non-medical, presumptive THC toxicosis from a private primary care practice and a private after-hours emergency practice were reviewed and summarized. Five of six cases were treated exclusively with transmucosal CBD (0.4–2.6 mg/kg); one case also received injectable anti-emetic therapy. Lethargy and ataxia noticeably improved and all additional clinical signs resolved within 45 min of treatment in five of six cases. No further follow-up measures for THC toxicosis were required in any case; one case required additional follow-up for presumably unrelated gastrointestinal distress. This is the first report of treatment of canine THC toxicosis by administration of CBD. The use of transmucosal CBD-infused dissolving sheets resulted in expedient resolution of clinical signs in a minimally invasive manner that is accessible to both clients and veterinary practitioners.

## 1 Introduction

Massive increases in cases of marijuana toxicosis have been reported in recent years (1–4), typically occurring through ingestion of the owner’s supply or secondary smoke inhalation (5). Toxicological effects are largely attributed to delta-9-tetrahydrocannabinol (THC) and severity is dependent upon dose and route of administration (6). The “classic” clinical presentation in dogs includes depression or ataxia and dribbling urine (7). Most commonly, diagnosis of THC toxicosis is made based on anamnesis and clinical signs; a positive urine drug-screening test can be supportive (5). Treatment of THC toxicosis has relied upon supportive care and minimizing toxin absorption (1, 4). Recovery from clinical signs can take between 30 min and 4 days (8). The prognosis is typically fair with early and aggressive treatment, though severe disease and fatalities are possible (4, 5, 9).

Barriers to managing cases of THC toxicosis include client-specific factors [e.g., cost of emergency care, fear of judgment (particularly where possession or use of THC is illegal)] and veterinary-specific factors (e.g., murky and evolving regulations in veterinary use of cannabis-related products) (4, 10–12). Care plans that incorporate less costly treatments and minimize intensive monitoring, when aligned with patient, client, and veterinary considerations, can expand the spectrum of care and mitigate the effects of some of these barriers (13). The aims of this case series are to describe the clinical and diagnostic findings and outcomes for six dogs with non-medical, presumptive THC toxicosis treated with CBD, and the use of a transmucosal dissolving sheet for CBD delivery in these patients.

## 2 Materials and methods

**Figure 1:**
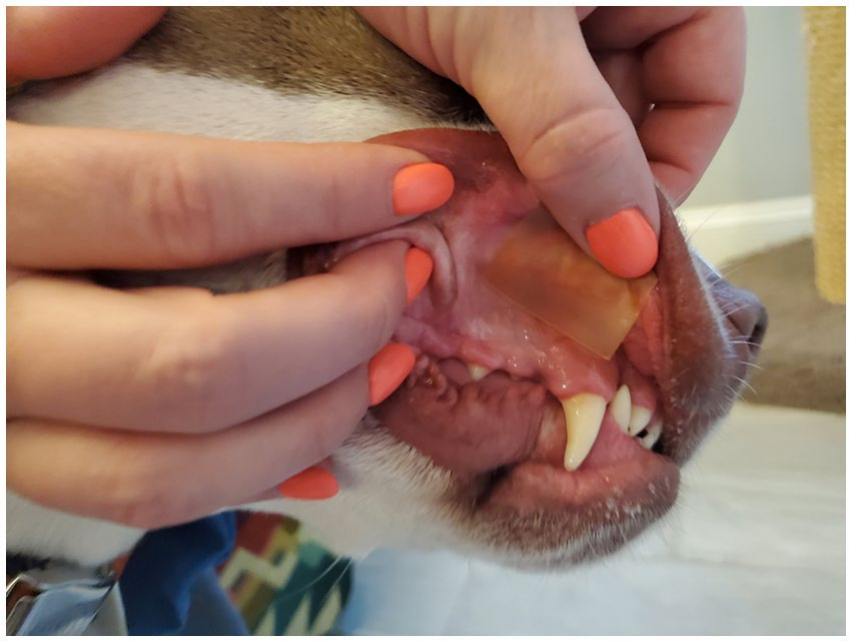
Administration of a transmucosal CBD-infused dissolving sheet.

Medical records from a private primary care and after-hours emergency practice in a suburban community of Jacksonville, FL between December 2023 and February 2024 were reviewed retrospectively. Records that included dogs with presumptive or known ingestion of THC prior to presentation treated with CBD-infused dissolving sheets (SimpliSolve, CBD Vet Products, Jacksonville, FL) were included. CBD was administered via transmuscosal application as described in product directions (Figure 1). Diagnosis of THC toxicity was determined by the attending veterinarian based on anamnesis (including owner reports of access to, suspected, or known ingestion of THC) in combination with clinical findings. Client communication records after presentation were reviewed for case follow-up post-discharge.

## 3 Case descriptions

### 3.1 Case 1

An 18.8 kg, 3-year-old spayed female Pitbull mix was presented for ataxia, swaying while sitting, and ptyalism 4–5 h prior to presentation. She urinated in the house and vomited once; vomitus contained a small piece of cloth. The owners suspected dietary indiscretion noting there was marijuana in the household. Three other canine housemates were unaffected. On physical examination, the patient was anxious and exhibited increased sensitivity to light and sound. Heart rate and respiratory rate were elevated at 120 beats per minute and 40 respirations per minute; body temperature was 99.4 degrees F (37.4 degrees C). She exhibited severe ataxia with crossing over of the hind limbs and mild urinary incontinence (i.e., dribbling). All other physical examination findings were unremarkable and the patient had no known or reported previous contact with THC-containing products.

The veterinarian determined the historical and clinical findings to be consistent with ingestion of an unknown quantity of THC and made a presumptive diagnosis of THC toxicosis. After owner consultation, the patient was treated with 10 mg (0.5 mg/kg) of CBD via administration of a CBD-infused transmucosal dissolving sheet and continuously monitored. Approximately 45 min after CBD administration, urine dribbling ceased, light and sound sensitivity resolved, and only a very mild ataxia remained. The patient was discharged and the owners were instructed to monitor for changes in gum color, loss of appetite, vomiting, diarrhea, panting or dyspnea, and lethargy; no additional follow-up information was available.

### 3.2 Case 2

A 24.5 kg, 8-year-old spayed female mixed breed dog was presented for lethargy, ataxia, and flinching at sudden movements beginning approximately 5 h prior to presentation. The owners suspected dietary indiscretion noting there was marijuana in the household. On physical examination, there was moderate dark brown-yellow debris in each external ear canal and she exhibited ataxia and increased sensitivity to light and sound. Heart rate and respiratory rate were 88 beats per minute and 16 respirations per minute; body temperature was 97.8 degrees F (36.5 degrees C). All other physical examination findings were unremarkable. Otic cytology was consistent with bilateral Malassezia otitis externa. This patient had no known or reported previous contact with THC-containing products.

The veterinarian determined the historical and clinical findings to be consistent with ingestion of an unknown quantity of THC and made a presumptive diagnosis of THC toxicosis. After owner consultation, the patient was treated with 10 mg (0.4 mg/kg) of CBD via administration of a CBD-infused transmucosal dissolving sheet and, due to non-specific clinical concerns of nausea, 245 mg maropitant citrate (Cerenia^®^, Zoetis, Inc., United States) intravenously. Approximately 45 min after CBD administration, light and sound sensitivity resolved, lethargy and ataxia were improved but still evident. The patient was discharged with topical therapy for otitis and instructions to seek re-evaluation if clinical signs worsen or do not resolve.

Approximately 22 h later, the patient was re-presented for anorexia and hematochezia. Lethargy and ataxia were further improved but still present. Heart rate and respiratory rate were 110 beats per minute and 20 respirations per minute; body temperature was 100.3 degrees F (37.9 degrees C). Physical examination findings and complete blood count were within normal limits. Blood chemistry analysis indicated decreased alkaline phosphatase (14 U/L; Ref 23-212) and elevated total bilirubin (1.1 mg/dL; Ref <0.9); all other values were within normal limits. A presumptive diagnosis of acute hemorrhagic diarrheal syndrome unrelated to THC ingestion was made and the patient was treated with crystalloid fluids subcutaneously. The patient was clinically stable and discharged with probiotics, mirtazapine, dietary recommendations, and instructions to follow-up with their primary care veterinarian in 1 week.

### 3.3 Case 3

A 4.9 kg, 5-month-old intact male French Bulldog was presented for sudden onset of lethargy after ingestion of an unknown amount of marijuana flower witnessed by the owner approximately 1 h prior to presentation. On physical examination, the patient was quiet, alert, and responsive. Mild stenosis of the nares was present. The cranial abdomen was distended and firm on palpation. The patient was ataxic and had head tremors. All other physical examination findings were within normal limits. Three-view abdominal radiographs were obtained and demonstrated a large amount of ingesta within the stomach. All other findings were within normal limits and this patient had no known or reported previous contact with THC-containing products.

The veterinarian determined the historical and clinical findings to be consistent with ingestion of an unknown quantity of THC and made a presumptive diagnosis of THC toxicosis. After owner consultation, the patient was treated with 2.5 mg (0.5 mg/kg) of CBD via administration of a CBD-infused transmucosal dissolving sheet. Approximately 45 min after treatment, ataxia and tremors resolved, the patient was discharged, and the owners were instructed to monitor for changes as described for case 1. Upon follow-up 18 h after presentation, the owners reported return of normal activity level and complete resolution of neurologic signs.

### 3.4 Case 4

A 3.9 kg, 4-month-old spayed female miniature Dachshund was presented for shaking and lethargy approximately 2 h after ingestion of ½ of a 100 mg THC gummy. On physical examination, the patient was anxious, hyper-reactive to light and sound, ataxic, and there was mild urinary incontinence (i.e., dribbling urine). Heart rate and respiratory rate were 130 beats per minute and 20 respirations per minute; rectal temperature was 101.6 degrees F (38.6 degrees C). All other physical examination findings were unremarkable and this patient had no known or reported previous contact with THC-containing products.

The veterinarian determined the historical and clinical findings to be consistent with ingestion of THC and made a presumptive diagnosis of THC toxicosis. After owner consultation, the patient was treated with 5 mg (1.3 mg/kg) of CBD via administration of a CBD-infused transmucosal dissolving sheet. Due to the high dose of THC ingested (12.8 mg/kg) and lack of immediate clinical response, an additional 5 mg of CBD was administered 20 min after the initial dose. Approximately 90 min after the first dose of CBD, mild ataxia was present while all other signs were resolved. The patient was discharged and the owners were instructed to monitor for changes as described for case 1. Upon follow-up communication approximately 16 h after presentation, the owners reported return of normal activity level and resolution of clinical signs.

### 3.5 Case 5

A 7.3 kg, 2-year-old neutered male Dachshund was presented for ingestion of an unknown quantity of THC edibles. Approximately 2 h prior to presentation, the owners noted ataxia, lethargy, and a single instance of vomiting. On physical examination, the patient was anxious, hyper-reactive to light and sound, exhibited ataxia, and mild urinary incontinence (i.e., dribbling urine). Heart rate and respiratory rate were elevated at 120 beats per minute and 40 respirations per minute; rectal temperature was 101.2 degrees F (38.4 degrees C). All other physical examination findings were unremarkable and this patient had no known or reported previous contact with THC-containing products.

The veterinarian determined the historical and clinical findings to be consistent with ingestion of an unknown quantity of THC and made a presumptive diagnosis of THC toxicosis. After owner consultation, the patient was treated with 5 mg (0.7 mg/kg) of CBD applied via administration of a CBD-infused transmucosal dissolving sheet. Approximately 40 min after CBD administration, hyperreactivity, ataxia, and urinary incontinence resolved and the patient was discharged. The owners were instructed to monitor for changes as described for case 1. Upon follow-up communication approximately 12 h after presentation, the owners reported continued lethargy and resolution of other clinical signs.

### 3.6 Case 6

A 5.0 kg, 10-year-old neutered male Jack Russell Terrier mix was presented for acute onset of vomiting, ataxia, and dribbling urine beginning approximately 3 h prior to presentation. The owners suspected dietary indiscretion noting there was marijuana in the household. On physical examination, the patient was quiet, alert, responsive, and anxious. He was hyper-reactive to light and sound and exhibited ataxia. Heart rate and respiratory rate were 102 beats per minute and 42 respirations per minute; rectal temperature was 102.0 degrees F (38.8 degrees C). All other physical examination findings were unremarkable and this patient had no known or reported previous contact with THC-containing products.

The veterinarian determined the historical and clinical findings to be consistent with ingestion of an unknown quantity of THC and made a presumptive diagnosis of THC toxicosis. After owner consultation, the patient was treated with 5 mg (1 mg/kg) of CBD via administration of a CBD-infused transmucosal dissolving sheet. Approximately 45 min after CBD administration, all clinical signs resolved and the patient was discharged. The owners were instructed to monitor for changes as described for case 1. Upon follow-up communication approximately 28 h after presentation, the owners reported return of normal activity level and resolution of clinical signs.

**Figure 2:**
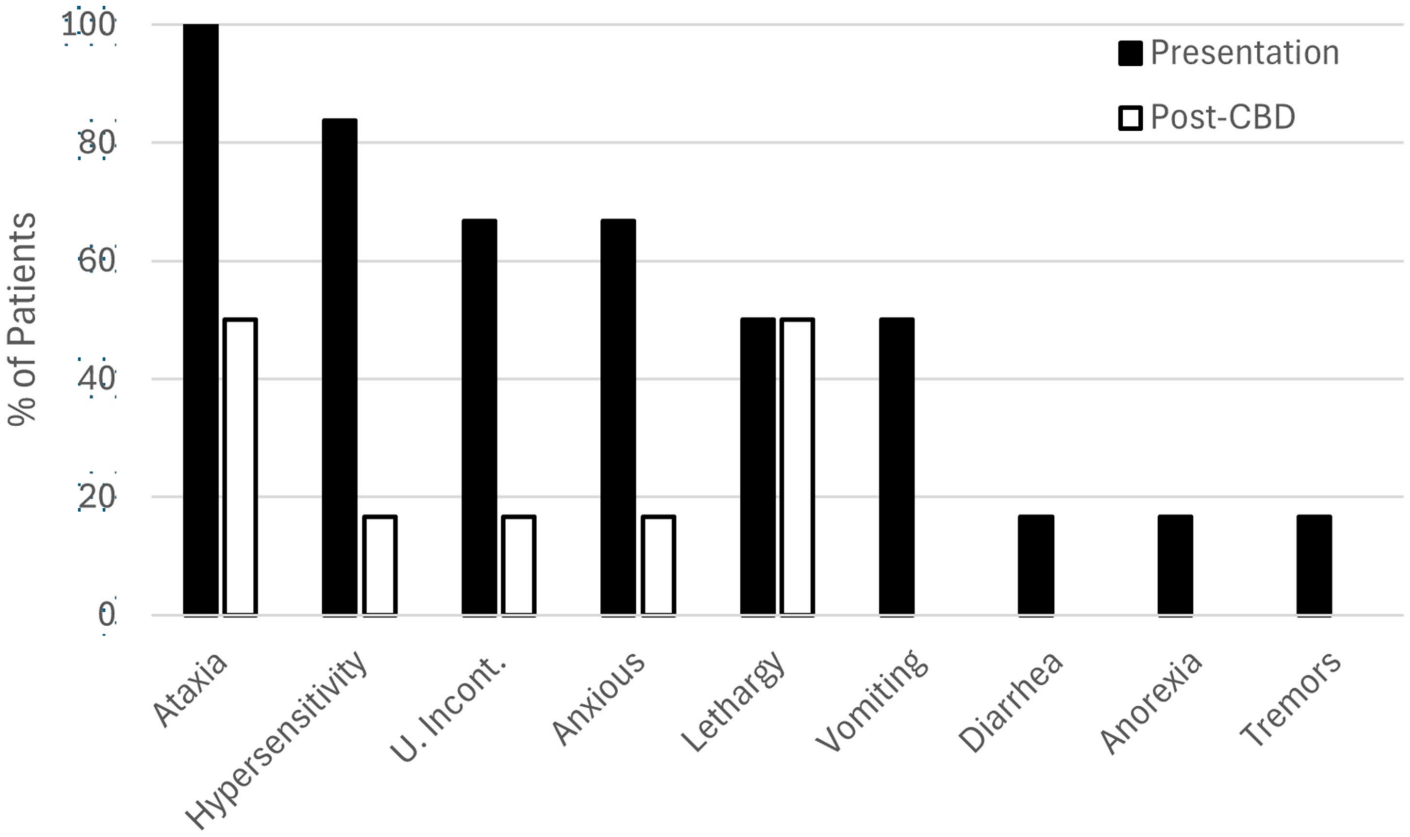
Clinical findings in six cases of canine THC toxicosis at presentation (solid bars) and 45 minutes after administration of transmucosal CBD-infused dissolving sheets (open bars).

A per-case summary of clinical findings is presented in Table 1 and Figure 2, as a percentage of patients. In this series, 3 dogs had known exposure to THC via ingestion of marijuana flower, THC gummies, or marijuana edibles. Cases of highly suspected exposure were via ingestion of undisclosed marijuana products. The approximate dose of ingestion could be calculated for one case (case 4, 12.8 mg/kg). Treatment of THC toxicosis was conducted via administration of CBD at doses ranging from 0.4–2.6 mg/kg. Presenting clinical signs were noticeably reduced in five of six cases within 45 min of CBD administration.

**Table 1:** Clinical findings in six individual cases of canine THC-toxicosis at presentation (✓) and 45 min after administration of CBD-infused transmucosal dissolving sheets (+).

Clinical sign	Case 1	Case 2	Case 3	Case 4	Case 5	Case 6
Ataxia	✓+	✓+	✓	✓+	✓	✓
Hypersensitivity	✓	✓		✓+	✓	✓
Urinary incontinence	✓			✓+	✓	✓
Anxiety	✓			✓+	✓	✓
Lethargy		✓+	✓+		✓+	
Vomiting	✓				✓	✓
Diarrhea		✓				
Anorexia		✓				
Tremors			✓			

## 4 Discussion

To the authors’ knowledge, this is the first report describing CBD administration to counteract the clinical effects of THC toxicosis and to describe the administration of transmucosal CBD-infused dissolving sheets in dogs. In this case series, clinical signs of toxicosis were completely resolved for three of six cases within 28 h of CBD administration; presenting clinical signs were noticeably reduced in five of six cases within 45 min. CBD administration was well-tolerated with no reports of challenges administering the transmucosal dissolving sheet or adverse effects.

Partly attributed to increasing legalization of marijuana products (1–5), exposure in dogs is typically through ingestion of the owner’s supply or secondary smoke inhalation (5) as is consistent with the cases reported here. Clinical signs have been reported for THC doses as low as 0.3 mg/kg (14). In the cases reported here, the dose of ingestion could only be estimated in case 4 at approximately 12.8 mg/kg. It is possible that the edible gummy ingested in this case contained additional compounds that potentiated the effects of THC or the labeled amount of THC in the product was inaccurate (15, 16). These factors may have necessitated the additional dose of CBD and prolonged the abatement of clinical signs as compared to the other cases in this series.

The clinical signs observed in this case series were limited to those described in Table 1 and Figure 2 and are consistent with the most common clinical scenarios presented to practitioners (4, 7). However, a variety of additional clinical signs have been reported in association with THC toxicosis, including: neurological (e.g., ataxia, proprioceptive deficits/delays, tremors, lethargy, an- or hyperesthesia, disorientation), ocular (e.g., mydriasis), gastrointestinal (e.g., ptyalism, vomiting, anorexia), sound or light sensitivity, inappropriate urination (i.e., urinary incontinence), hypothermia, bradycardia, and increased serum alkaline phosphatase activity (17–21). Depression or ataxia while dribbling urine is common with clinical signs appearing as soon as 30 min after ingestion or delayed by several hours (7, 22). Although the specific interval between THC ingestion and the initiation of clinical signs was not known in the cases reported here, patients were presented 1–5 h after their development. Differential diagnoses for these clinical signs include ingestion of illicit hallucinogens, alcohol ingestion (including ethylene glycol), overdosage of sedative pharmaceuticals (e.g., opioids, benzodiazepines), macrocyclic lactone toxicity, and in some cases, intervertebral disc disease or head trauma (23). Most commonly, diagnosis of THC toxicosis is made based on anamnesis and clinical signs as was the case for each of the patients reported here. The quick resolution and/or improvement of the majority of presenting clinical signs of toxicosis after CBD treatment and in the absence of other therapies in five of these six cases further supports their diagnoses. The use of human urine drug-screening tests in animals is possible in the clinical setting but sample handling errors are common and sensitivity is low (5); for these reasons such tests are not stocked or otherwise relied upon as diagnostic tools in the clinics that provided cases for this report.

THC toxicosis treatment has been based on supportive care and minimizing toxin absorption and, dependent upon timing and dose of ingestion, may include: emesis and/or administration of anti-emetics, gastric lavage, activated charcoal administration, intravenous lipid emulsion, thermal support, blood pressure monitoring, and sedation (1, 4). Recovery from clinical signs can take anywhere from 30 min to 4 days (8). Outpatient treatment is most common; however, hospitalization for <6 to 48 h may be indicated (4). The prognosis is typically fair, particularly with earlier and more aggressive treatment, however severe disease and fatalities are possible and of particular concern with ingestion of synthetic cannabinoids or marijuana concentrates (4, 5, 9). Details about the specific formulation of THC that was ingested in cases 1, 2, 5, and 6 were not available.

CBD directly antagonizes the excitatory effects and potentiates the depressant effects of THC in mice, rabbits, rats, and humans (24, 25). The mechanism of this action is through displacement of THC from its plasma protein binding sites by CBD (26). These actions may be impacted by the dose, timing, and route of administration of both compounds which may explain the partial or delayed improvement in some clinical signs in this case series (i.e., lethargy). In one report, high doses of oral CBD inhibited the metabolism of oral THC, resulting in increased undesirable effects (e.g., anxiety, sedation, tachycardia, psychomotor performance) in humans (26); it is unknown, but plausible that similar effects could be seen in dogs with variations in THC + CBD dosing and/or route of CBD administration. In the authors’ experience, the potential application of CBD to antagonize THC is not widely known among veterinary practitioners. CBD is not psychotropic, however, small amounts of THC may be present and products may not have undergone quality control assurance (thus containing higher levels of THC than labeled or permitted) (27). For these reasons, veterinarians hesitate to use some CBD products in THC toxicity cases.

The active ingredient in the product used in these cases is CBD from broad spectrum hemp. Each sheet contains 10 mg CBD evenly distributed throughout a dissolving thin film, allowing for dose-splitting per sheet. Product labeling suggests doses based on body weight: 2.5 mg for dogs ≤9 kg, 5 mg for >9–22.7 kg, and 10 mg for >22.7 kg. The product is labeled for general endocannabinoid system support in both dogs and cats at a retail cost of about $2 per 10 mg sheet. Content verification is confirmed through individual batch testing by a third-party laboratory. In contrast to conventional oral solid formulations, thin films typically offer significantly increased drug bioavailability owing to avoidance of first-pass hepatic metabolism, rapid mucosal absorption, and increased solubility and rate of dissolution (28). The pharmacokinetics of the transmucosal formulation used in these cases has been independently evaluated. Serum samples from a single adult cat and two adult dogs suggest peak concentration is reached 30–60 min after administration with rapid decline after 2 h (data on file). Finally, the ease of transmucosal administration in anxious animals supports practitioners employing low-stress handling techniques and may be more desirable than alternative formulations when oral treatments are contraindicated (e.g., vomiting).

Inability to afford emergency services is a leading barrier to accessing veterinary care in both the United States and Canada (11, 12). A survey of veterinarians in the United States and Canada reported treatment costs for THC toxicosis of up to $2,000 (4). While the most common cost was <$500 in that report, a recent pet owner survey found that a “surprise” vet bill of $499 or less would cause 28% of those surveyed to go into debt (29). In the clinics providing cases for this report, the client cost of a physical examination and administration of CBD, in the absence of additional diagnostics, was $150. These care plans were relatively low cost and avoided hospitalization and its associated pressures on staff time and clinic operations. It is noteworthy that the diagnosis in each of the cases reported here relied upon anamnesis, observational examination, and ultimately response to treatment, rather than physical findings or laboratory diagnostics. Furthermore, in five out of the six cases, physical diagnostics, instrumental monitoring, or prescription medications were not clinically indicated as determined by the attending veterinarian. These findings suggest that additional models of care (e.g., telemedicine) may be appropriately and safely employed in cases of THC ingestion where legally permissible, thus further mitigating barriers to accessing care in similar scenarios.

There are several limitations to the extrapolation of the clinical findings of the cases reported here to the wider population of canine THC toxicosis cases. First, although a diagnosis of THC toxicosis is typically based on anamnesis and clinical signs as has been discussed, THC ingestion cannot be confirmed in all cases. Although some differentials can be reliably ruled out by physical examination, ingestion of other toxins, particularly illicit compounds, were unlikely to be detected by the case workups conducted. While possession and use of CBD products is legal in the state Florida, intoxication with other illicit substances could have similar clinical presentations and owners may have been less willing to discuss access to these compounds with the attending veterinarians. Second, partial or complete resolution of clinical signs occurred rapidly after transmucosal CBD administration in each of these cases consistent with the known pharmacokinetics of the specific product used. However, dosage studies have not been performed and both under- and over-dosing relative to the amount needed to displace THC from its binding sites in the patients with partial and complete resolution respectively, as well as potentiation of THC adverse effects similar to those reported in humans (26), was possible. Notably, the dose determined by the attending veterinarian(s) in these cases was higher than product labeling (for general endocannabinoid support) in four of the six cases, based largely on subjective assessment of the severity of clinical signs and experience with the CBD product used. Additionally, the timeline of clinical recovery in each of these cases is within the known period of recovery from THC toxicosis in the absence of specific medical therapy. Therefore, it is possible that the observed clinical regressions would have occurred naturally. Finally, the cases reported here were relatively mild at the time of presentation and inpatient care was not indicated. As there is little information in the scientific literature about the use of CBD to counteract THC toxicosis, the purported effects of transmucosal CBD in these cases might not be the same as those in more severe cases, those treated >5 h after the development of clinical signs, those treated with different dosages, or those treated by alternate routes of CBD administration. Additional randomized, prospective data is warranted to better describe the use of CBD to counteract the clinical effects of canine THC toxicosis, including dosing studies and measurement of the time to effect versus natural resolution.

## Data Availability

The original contributions presented in the study are included in the article/supplementary material, further inquiries can be directed to the corresponding author.
